# Proteomic Profiling of Bone Marrow Aspirates from Patients with Methotrexate- and Vincristine-Resistant B-Cell Acute Lymphoblastic Leukemia: A Retrospective Analysis

**DOI:** 10.3390/ph19081167

**Published:** 2026-07-26

**Authors:** Esli Janai Flores-Palma, Diana Laura Gonzalez-Tolentino, Sergio Encarnación-Guevara, Jeovanis Gil, Ramiro Alonso-Bastida, Angel Gabriel Martínez-Batallar, Mónica Virginia Saavedra-Herrera, Eloísa Ibarra-Sierra, Yazmín Gómez-Gómez, Berenice Illades-Aguiar, Olga Lilia Garibay-Cerdenares, Marco Antonio Leyva-Vázquez

**Affiliations:** 1 Laboratorio de Biomedicina Molecular, Facultad de Ciencias Químico Biológicas, Universidad Autónoma de Guerrero, Chilpancingo 39090, Guerrero, Mexico; 15168911@uagro.mx (E.J.F.-P.); 11082612@uagro.mx (D.L.G.-T.); yazmingomez@uagro.mx (Y.G.-G.); billades@uagro.mx (B.I.-A.); 2 Centro de Ciencias Genómicas, UNAM, Cuernavaca 62210, Morelos, Mexico; encarnac@ccg.unam.mx (S.E.-G.); rbastida@ccg.unam.mx (R.A.-B.); angelmb@ccg.unam.mx (A.G.M.-B.); 3 Department of Translational Medicine, Lund University, 22242 Lund, Sweden; jeovanis.gil_valdes@med.lu.se; 4 Instituto Estatal de Cancerología “Dr. Arturo Beltrán Ortega”, Acapulco 39570, Guerrero, Mexico; monisaav2003@yahoo.com.mx (M.V.S.-H.); comite.etica@cancerologiagro.gob.mx (E.I.-S.); 5 Secretaría de Ciencia, Humanidades, Tecnología e Innovación (SECIHTI), SECIHTI-Universidad Autónoma de Guerrero, Chilpancingo 39090, Guerrero, Mexico

**Keywords:** B-ALL, methotrexate, vincristine, proteomics, chemoresistance

## Abstract

**Background**: B-cell acute lymphoblastic leukemia (B-ALL) is the most frequent malignancy of childhood; worldwide, 487,294 new cases and 305,405 deaths were reported in 2022, including more than 5000 new cases in Mexico. Chemotherapy, delivered in induction, consolidation and maintenance phases, remains the mainstay of treatment, yet 10–20% of patients relapse after induction because of chemoresistance, whose molecular basis is still incompletely understood. **Methods**: This retrospective study aimed to identify proteins associated with resistance to vincristine (VCR) and methotrexate (MTX) administered during the induction phase, using LC–MS/MS proteomics and bioinformatic analysis of treatment-naive bone marrow aspirates from responders and nonresponders. **Results**: Nonresponders showed a distinct proteomic profile, with deregulated processes converging on cytoskeletal structure and dynamics, nucleic acid metabolism, DNA repair and RNA processing. Within these processes, thymidine phosphorylase (TYMP) and gelsolin (GSN) emerged as differentially expressed proteins, both consistently overexpressed in nonresponders at the individual-patient level. **Conclusions**: We conclude that cytoskeletal remodeling and nucleotide metabolism are prominent features of intrinsic chemoresistance in pediatric B-ALL, and that TYMP and GSN represent candidate biomarkers of nonresponse to VCR- and MTX-based induction that warrant validation by orthogonal methods in independent, adequately powered cohorts.

## 1. Introduction

B-ALL is a clonal hematological malignancy that arises when a B-lymphoid progenitor acquires genetic lesions, chromosomal translocations that generate oncogenic fusion transcription factors or kinases, aneuploidy, and mutations in genes governing lymphoid differentiation that arrest maturation at the precursor stage and confer aberrant self-renewal and a survival advantage [1]. The resulting lymphoblasts expand clonally in the bone marrow, displacing normal hematopoiesis. This multistep oncogenetic process frequently begins in utero with a pre-leukemic founding clone, which requires additional postnatal genetic hits to progress to overt leukemia, and it accounts for the relatively high incidence of the disease in children [2,3].

In 2021, the worldwide incidence of B-ALL increased from 98,149 to 103,727 cases and in prevalence from 237,056 to 386,813 cases [2]. In Mexico, acute leukemias are the leading cause of disease mortality in children under 19 years of age [4].

Approximately 80–85% of childhood leukemias are B-cell precursors, in which lymphoblasts lack the IgH heavy chain of the B-cell receptor (BCR), thereby interrupting their maturation [5,6]. It has also been reported that genetic alterations such as translocations that lead to the fusion of transcription factors such as ETS variant transcription factor 6 and Runt-related transcription factor 1 (ETV6::RUNX1) (25% in children), transcription factor 3, pre-B-cell leukemia transcription factor 1 (TCF3::PBX1) (5% in children), paired box protein Pax-5, ETS variant transcription factor 6 (PAX5::ETV6) (8–10%) or the fusion of genes that function as signal transducers such as breakpoint cluster region protein and tyrosine-protein kinase ABL1 (BCR::ABL1, 10–15% in children) [7,8] promote lymphocyte development, hematopoiesis [3], and the survival and proliferation of immature cells [9,10,11].

There are four standard treatment options for childhood B-ALL: chemotherapy, radiotherapy, chemotherapy with stem cell transplantation (most commonly used in relapse), and targeted therapy. Chemotherapy is administered in three main phases: induction, consolidation (intensification), and maintenance. Each phase aims to eradicate residual leukemic blasts [12]. The drugs administered during these stages are methotrexate, vincristine [13], glucocorticoids, anthracyclines [14], asparaginase, cytarabine [15], L-asparaginase, and mercaptopurine [16].

In Guerrero, children with B-ALL are treated with vincristine and methotrexate during the induction–consolidation phases; however, more than 20% of these patients experience relapse (nonresponse to chemotherapeutic treatment) [17], with chemoresistance being among the leading causes of mortality [18]. In B-ALL, several mechanisms associated with nonresponse to treatment have been evaluated in cell lines, animal models or patient samples, in which genetic alterations such as mutations in paired box protein Pax-5 (PAX5) have been described [19]. Moreover, overexpression of drug efflux transporters—such as ATP binding cassette subfamily G member 2 (ABCG2) and ATP-dependent translocase ABCB1 (MDR1) [20,21]; activation of anti-apoptotic pathways through caspase 3 (CASP3), caspase 8 (CASP8) [22], B-cell lymphoma-extra large (BCL-XL) [23] and apoptosis regulator Bcl-2 (BCL2) [24]; and epigenetic changes, such as hypermethylation of tumor suppressor genes through histone deacetylase 3 (HDAC3) [25] and dysregulation of microRNAs such as miR24 [26], miR27-a [27], miR-128 [28] and miR-125b [29]—has been associated with relapse in patients with B-ALL [6], including changes in the bone marrow microenvironment that contribute to disease persistence and relapse [30], such as overexpression of integrin alpha-4 (ITGA4) and integrin alpha-6 (ITGA6) [31].

In B-ALL, omics techniques have been used to advance our understanding of the disease; this information has been crucial for diagnosis, prognosis, and treatment, enabling more precise and personalized therapeutic interventions [32]. Transcriptomic analyses in adults and children with B-ALL have enabled the identification of distinct B-ALL subtypes, incorporated into conventional cytogenetic classification, and reinforced the role of genes such as PAX5, which is involved in leukemogenesis [33,34]. In adults with B-ALL, using transcriptomics and mutation profiles, a classification system with therapeutic implications (COMBAT) was created to assess patient survival or response to chemotherapy based on the presence of surface antigens associated with stem cell properties, as well as the infiltration of immune cells and endothelial cells [35]. Furthermore, using proteomics, transcriptomics, and pharmacoproteomics approaches in 49 pediatric ALL cell lines, a high-risk subgroup was identified, with MEF2D::HNRNPUL1 rearrangement as a potential biomarker for the response to anticancer drugs, such as the diacylglycerol analog bryostatin-1 [36]. Proteomics enables the identification of functional molecular changes in proteins that confer resistance to certain drugs in cancer cells. Some studies have used proteomics as an analytical tool in B-ALL and in T-ALL cell lines (CCRFE-CEM). Resistant subline CEM/VCR R and CEM/VLB100 proteins, such as β-tubulin, α-tubulin, actin, L-plastin, and lamin B1, among others, have been shown to be related to resistance to vinca alkaloids [37]. Moreover, in B-ALL xenografts resistant to VCR, proteins related to the actin cytoskeleton, such as gelsolin, ezrin, moesin, and tropomyosin, among others, have been identified [38].

Although transcriptomic and pharmacoproteomic frameworks have proven powerful for diagnostic and prognostic stratification, they have not been translated into a functional proteomic signature directly linked to clinical chemoresponse in treatment-naive pediatric patients. The molecular determinants of resistance specifically to vincristine and methotrexate, two cornerstone drugs of the induction phase and those used in our regional treatment protocol, have not been systematically characterized in clinically defined responder versus nonresponder pediatric cohorts at the time of diagnosis and pediatric B-ALL populations from Latin America, where induction failure and chemoresistance-related mortality remain disproportionately high. The novelty and strength of this work therefore reside in profiling the functional proteome of pediatric B-ALL blasts obtained directly from treatment-naive patients and stratified by their actual clinical response to vincristine and methotrexate, and in integrating unbiased proteomic discovery with bioinformatic analysis to recover both candidate biomarkers and the biological processes underlying chemoresistance, providing, to our knowledge, the first clinically anchored, proteomics-driven characterization of induction-phase chemoresistance in an under-represented Mexican pediatric cohort.

## 2. Results

### 2.1. Overview of Proteomic Analysis

**Figure 1:**
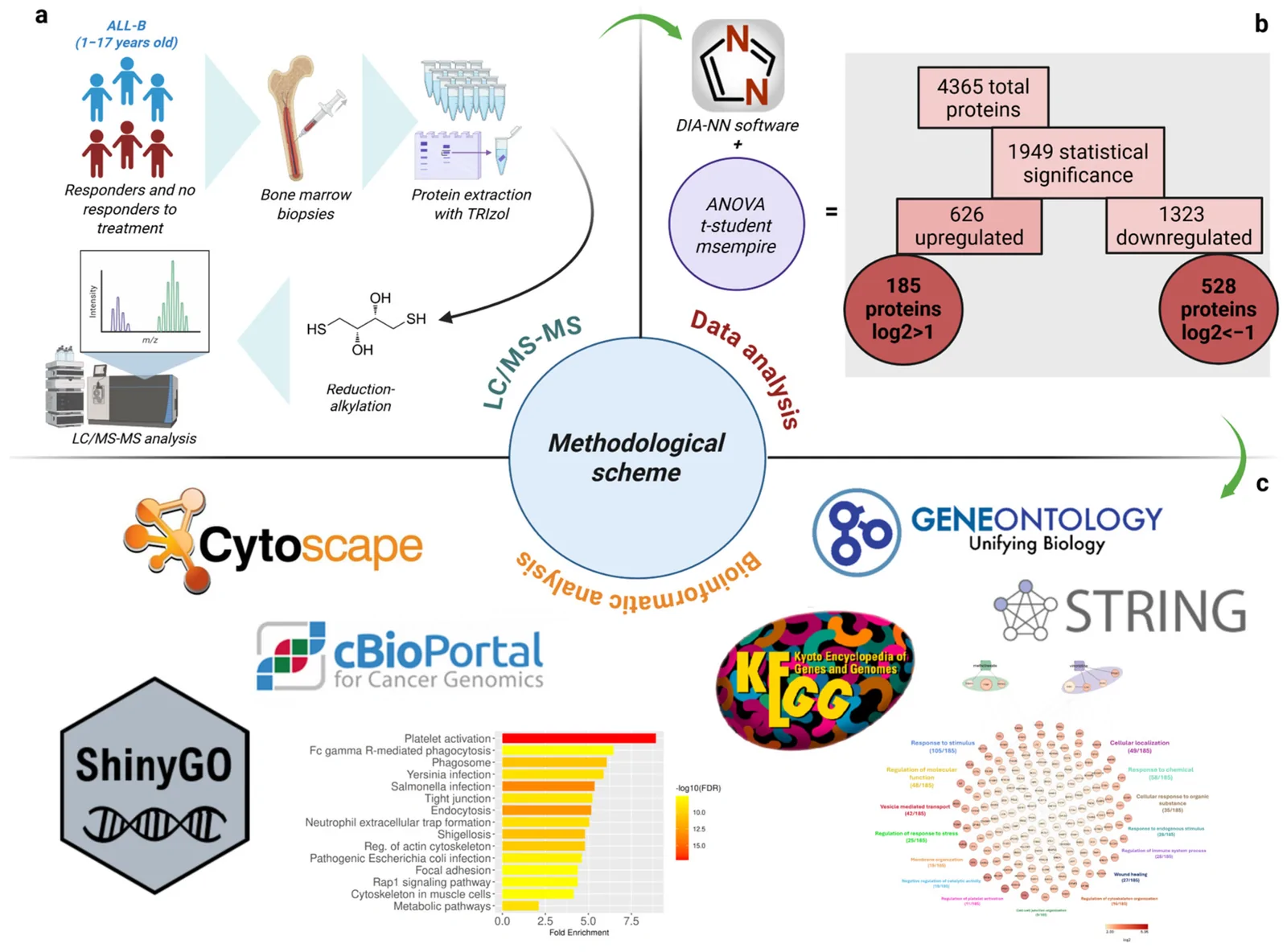
Methodological scheme of the study. (**a**) Description of the liquid chromatography analysis coupled to mass spectrometry. (**b**) Analysis of results using the MSEA statistical method, with *p* < 0.0001 considered statistically significant, and the total number of proteins obtained. (**c**) Bioinformatics programs for biological pathways, protein interactomes, and interactome editing. Created in BioRender. Flores, E. (2026) https://BioRender.com/h22m81v (accessed on 23 July 2026).

The overall experimental and analytical strategy employed for the identification of differentially expressed proteins (DEPs) associated with nonresponse to vincristine and methotrexate is outlined in Figure 1. In summary, bone marrow aspirates from responders and nonresponders were briefly cryopreserved, processed for protein extraction and tryptic digestion, and analyzed by LC–MS/MS under a single standardized DIA workflow. The resulting protein abundance matrices were subjected to differential expression and functional enrichment analyses (Figure 1a–c).

For the differential proteomic analysis, patients were stratified into responders and nonresponders to first-line chemotherapy. The responder group was defined as the reference condition for all two-group comparisons. The responders were operationally characterized by a minimal residual disease (MRD) level of ≤0.5%, representing the baseline posttreatment disease state and providing a clinically relevant baseline for comparison. Based on this definition, positive log2 fold change values indicate proteins overexpressed in nonresponders, whereas negative values correspond to proteins enriched in responders. This criterion was consistently applied across all downstream analyses to ensure a coherent and interpretable assessment of differential protein expression.

Initially, 10 samples per group were selected (n = 20). Following protein extraction, only 19 samples met the required quantification threshold (≥50 µg) for downstream processing, including reduction, alkylation, and enzymatic digestion. During LC–MS/MS analysis, adequate chromatographic separation could not be achieved for two samples, and these samples were therefore excluded. Consequently, 17 samples met all the quality control criteria and were included in the final analysis. Protein abundance data for all 4365 quantified proteins across these 17 samples are reported in the full proteomics dataset deposited in the ProteomeXchange Consortium (identifier PXD074132). Individual per-patient abundance values are additionally provided for the two proteins subsequently prioritized as candidate markers of nonresponse on the basis of the differential expression and pathway enrichment analyses described in Section 2.2, Section 2.3 and Section 2.4: gelsolin (GSN), an actin-severing protein involved in cytoskeletal remodeling, and thymidine phosphorylase (TYMP), an enzyme of the pyrimidine salvage pathway (Supplementary Table S2).

**Figure 2:**
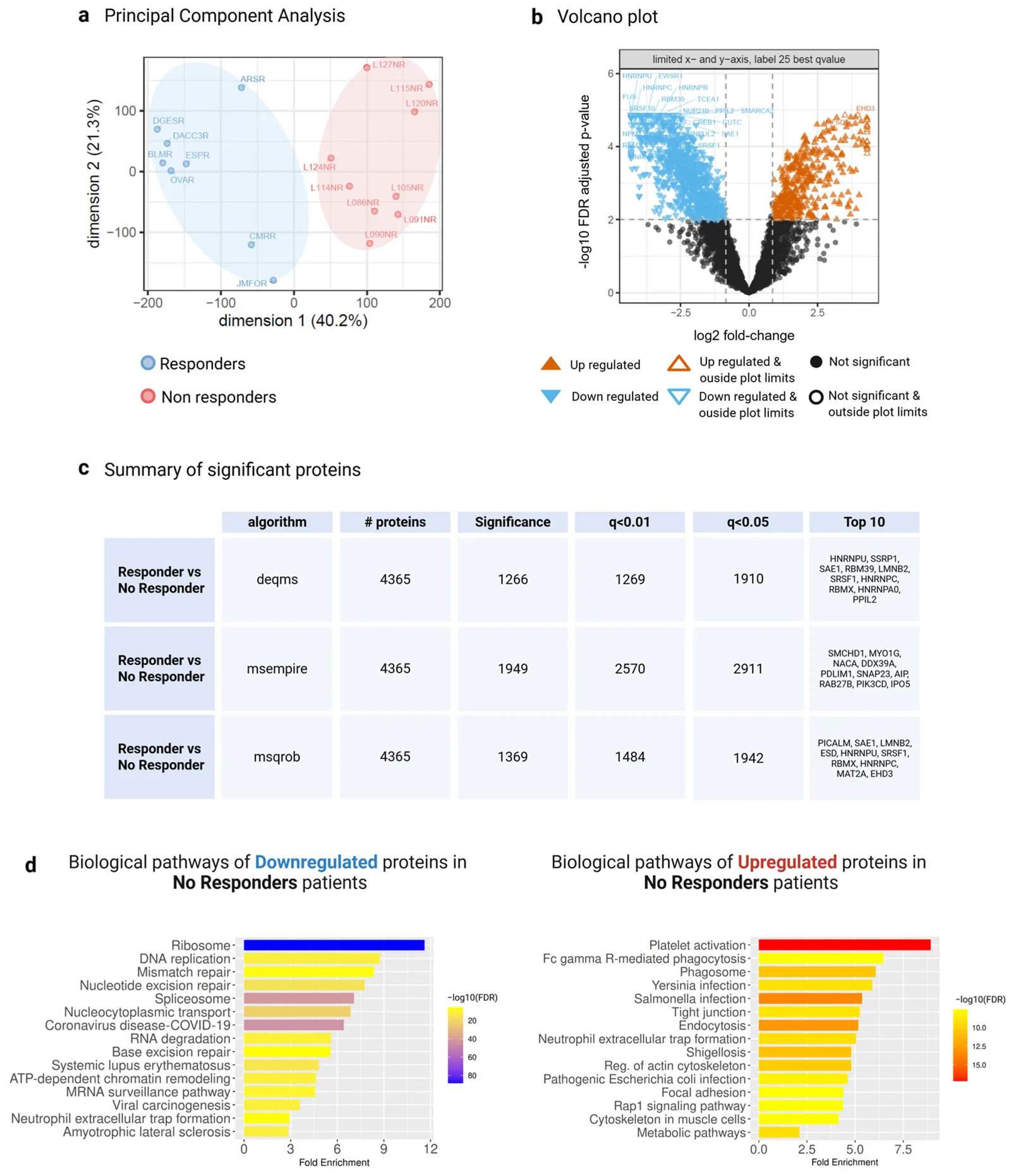
Proteomic changes and enriched biological pathways in the proteome of pediatric nonresponders to treatment. (**a**) PCA of the 9 nonresponders and 8 responders based on their protein expression profiles. PC1 and PC2 explain 40.2% and 21.3% of the variance, respectively. (**b**) Volcano plot of the 25 under- and overexpressed proteins in nonresponder children. (**c**) Significant proteins selected for analysis. (**d**) Biological pathways deregulated in the proteome of pediatric nonresponder patients. Created in BioRender. Flores, E. (2026) https://BioRender.com/h22m81v (accessed on 23 July 2026).

To explore the differences between protein abundance in responders and nonresponders to treatment, a principal component analysis (PCA) was performed, which revealed that the expression profiles of 1949 proteins in the two principal components (C1 and C2) explained more than 60% of the total variation, and the samples from nonresponders clustered separately from those from responders, suggesting consistent proteomic differences associated with chemoresistance (nonresponse) (Figure 2a). Volcano plot analysis of differential expression (Figure 2b) revealed that out of the 1949 differentially expressed proteins (DEPs) analyzed by MS-EmpiRe (Figure 2c), 1323 were underexpressed, and 626 were overexpressed (adjusted *p* value < 0.01). Pathway enrichment analysis of the DEPs revealed that underexpressed proteins were associated with processes such as DNA replication, nuclear–cytoplasmic transport, and RNA degradation. In contrast, overexpressed DEPs were associated with processes such as platelet activation, phagocytosis, tight junctions, and endocytosis (Figure 2d).

### 2.2. Underexpressed Proteins Interacting with Methotrexate and/or Vincristine

**Figure 3:**
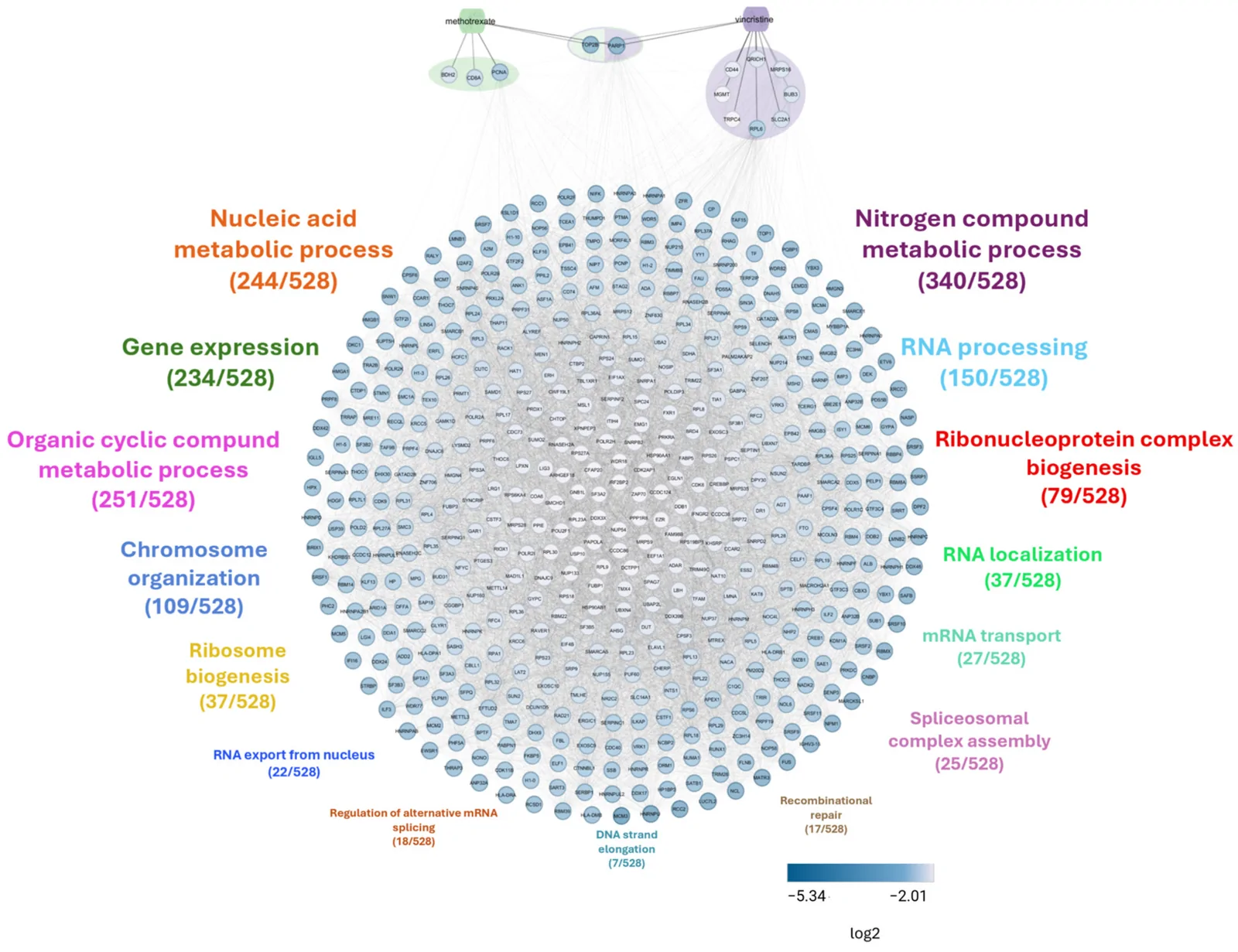
Protein–protein interactions between MTX and VCR. Protein–protein interactions of the 527 underexpressed proteins in pediatric nonresponders, where the color of the nodes indicates the log value. Edges indicate coexpression, experimental interaction, co-occurrence, or fusion.

Once the underexpressed DEPs were determined, protein–drug interaction maps were constructed to evaluate the probable physical, direct interactions with methotrexate and vincristine (confidence of 0.400). The analysis revealed 13 proteins with evidence of interaction with vincristine: CD44 antigen (CD44), transcriptional regulator QRICH1 (QRICH1), small ribosomal subunit protein bS16m (MRPS16), mitotic checkpoint protein BUB3 (BUB3), solute carrier family 2 (SLC2A1), large ribosomal subunit protein eL6 (RPL6), short transient receptor potential channel 4 (TRPC4) and methylated DNA–protein cysteine methyltransferase (MGMT). The proteins that showed evidence of interaction with methotrexate included dehydrogenase/reductase SDR family member 6 (BDH2), T-cell surface glycoprotein CD8 alpha chain (CD8A) and proliferating cell nuclear antigen (PCNA). DNA topoisomerase 2-beta (TOP2B) and poly [ADP-ribose] polymerase 1 (PARP1) interacted with both drugs. Functional enrichment analysis considering only proteins with log2 values between −2 and −5 revealed that underexpressed proteins (528) were associated with processes related to nucleic acid and cyclic organic compound metabolism, RNA processing, gene expression, and chromosomal organization, among others (Figure 3).

### 2.3. Overexpressed Proteins Interacting with Methotrexate and/or Vincristine

**Figure 4:**
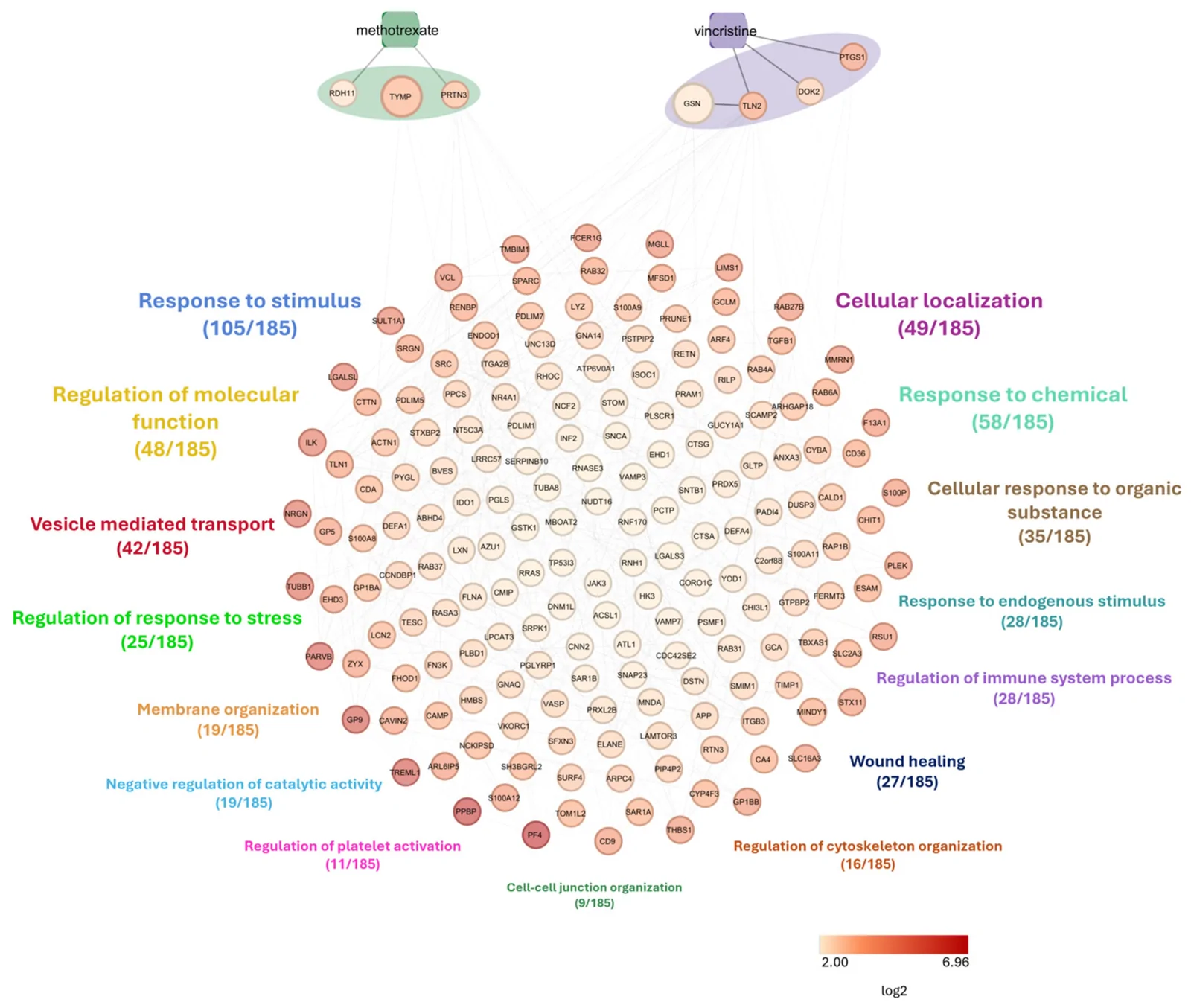
Protein–protein interactions between MTX and VCR. Protein–protein interactions of the 185 overexpressed proteins in pediatric nonresponders. The color of the nodes indicates the log value. Edges indicate coexpression, experimental interaction, co-occurrence, or fusion.

To identify overexpressed DEPs that interacted with methotrexate and/or vincristine, functional interactomes (confidence 0.400) were generated; the results revealed that the interactions with methotrexate included retinol dehydrogenase 11 (RDH11) and proteinase 3 (PRTN3); the interactions with vincristine included prostaglandin G/H synthase 1 (PTGS1), docking protein 2 (DOK2), and talin-2 (TLN2). The functional interactomes of the overexpressed DEPs, whose log2 values ranged from +2 to +6.9, were enriched for pathways such as stimulus response, subcellular localization, response to chemicals, and regulation of molecular function (Figure 4).

### 2.4. TYMP and GSN Are Overexpressed in MTX and VCR Nonresponders

**Figure 5:**
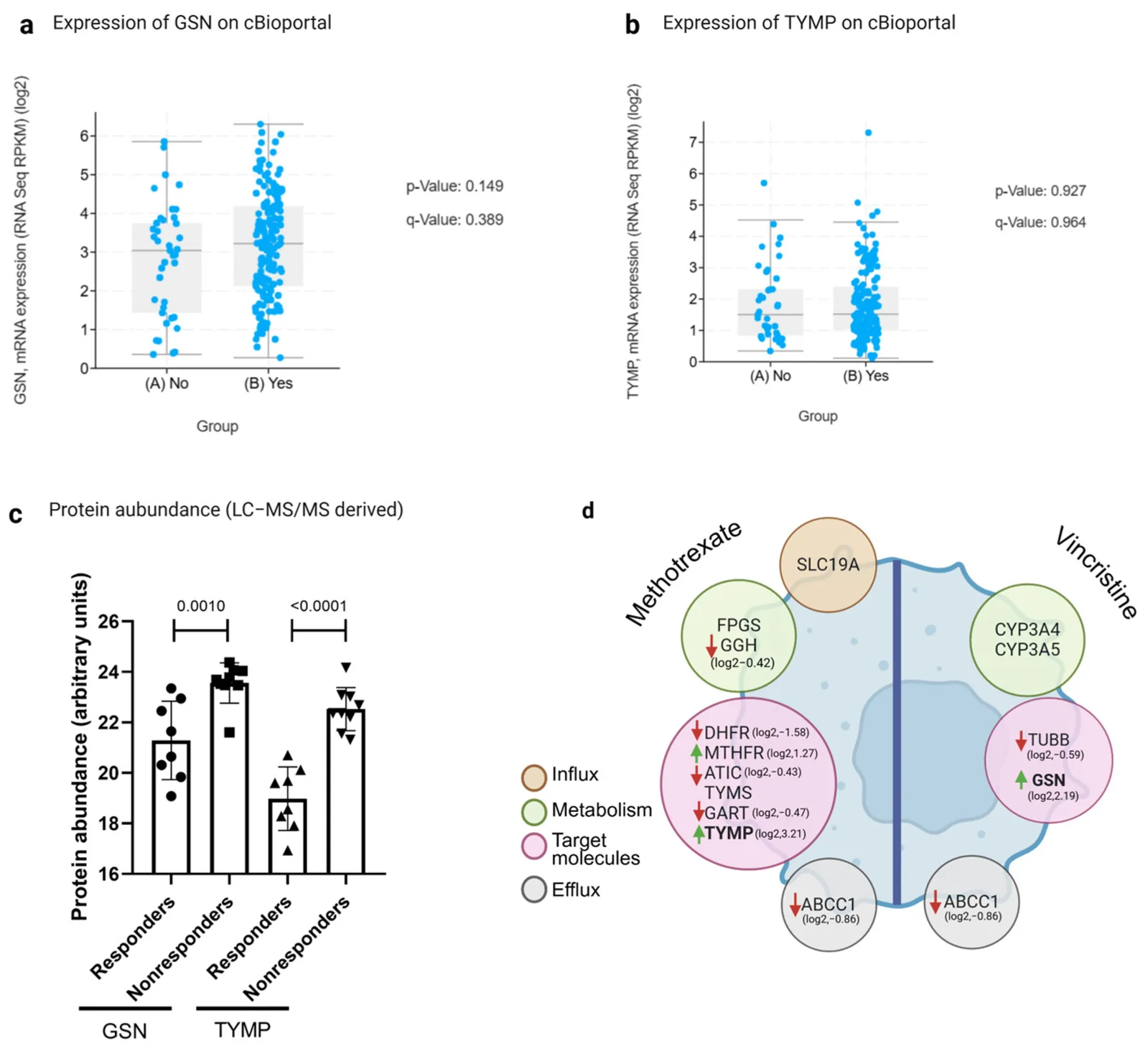
TYMP and GSN expression in children with B-ALL. (**a**,**b**) cBioportal datasets showing GSN and TYMP mRNA expression in children with B-ALL who did and did not experience relapse. (**c**) Relative abundance of proteins from LC-MS/MS from individual samples. (**d**) Summary of proteins involved in chemoresistance to MTX and VCR. The arrows in green and red represent the protein abundance in children nonresponders to treatment or fusion, respectively. Created in BioRender. Flores, E. (2026) https://BioRender.com/h22m81v (accessed on 23 July 2026).

To validate the differential expression identified in the proteomic data, two DEPs, TYMP (Log2 +3.21) and GSN (Log2 +2.19), were selected as candidate proteins based on the LC-MS/MS proteomic dataset and per-patient abundance analysis, given their differential expression between methotrexate/vincristine responders and nonresponders. Initially, the expression profiles of both genes were determined in a database of pediatric patients with relapsed and nonrelapsed B-ALL from the cBioPortal The Cancer Genome Atlas (TCGA dataset), comprising 1544 patients: 1271 (nonrelapsed) and 273 (relapsed). The data revealed that GSN (log2 ratio −0.38, *p* < 0.149) and TYMP (log2 ratio 0.02, *p* < 0.927) were differentially expressed, although not significantly (Figure 5a,b). The relative abundances per sample derived from the LC-MS/MS analysis were evaluated, revealing a significant difference between responders and nonresponders for GSN (*p* = 0.00010) and TYMP (*p* < 0.0001) (Figure 5c; individual per-patient values provided in Supplementary Table S2).

Finally, a model integrating the most important results was constructed, describing a group of proteins that are involved in chemoresistance (nonresponders) to methotrexate and vincristine, which are involved in processes such as transport for drug efflux and influx (reduced folate transporter (SLC19A1)) [39]), metabolism (cytochrome P450 family 3 subfamily A member 4 (CYP3A4)), and cytochrome P450 family 3 subfamily a member 5 (CYP3A5)) [40]. Some are molecular drug targets, such as dihydrofolate reductase (DHFR) [41], folylpolyglutamate synthase (FPGS), gamma-glutamyl hydrolase (GGH) [42], methylenetetrahydrofolate reductase (NADPH) (MTHFR) [43], bifunctional purine biosynthesis protein ATIC (ATIC), thymidylate synthase (TYMS), trifunctional purine biosynthetic protein adenosine-3 (GART), ABC transporter C family member 1 (ABCC1) [39], and tubulin beta chain (TUBB) [40]. Some of these proteins were identified as overexpressed (MTHFR and TYMP) or underexpressed (DHFR, ATIC, GART, TUBB, and ABCC1), suggesting that they could be potential targets for validation in nonresponder patients (Figure 5d).

To benchmark the discovery findings against previously reported resistance mechanisms, the dataset was interrogated for a curated panel of canonical mediators (Supplementary Table S4). Several were recovered as DEPs in the expected direction: DHFR, ATIC, and TUBB (underexpressed), MTHFR (overexpressed), and the adhesion integrins ITGA2B and ITGB3 (overexpressed), supporting the internal validity of the proteomic workflow, whereas low-abundance transporters and transcription factors (ABCB1, ABCG2, PAX5) were not detected under a single-shot DIA strategy.

## 3. Discussion

B-ALL is a disease with a relatively high frequency in children, where relapse, which occurs in 20% of treated patients, is among the leading causes of death [44,45]. However, why patients do not respond well to treatment remains unclear. In the present study, a proteomic analysis was performed on bone marrow aspirates from pediatric patients diagnosed with B-ALL, with clinical data indicating nonresponse to methotrexate and vincristine during the induction phase.

The functional analysis of nonresponse to MTX- and VCR-based induction identified coherent sets of up- and downregulated processes rather than isolated protein changes. These processes are directly relevant to the pharmacology of both drugs, since they involve the cellular machinery that MTX and VCR target, nucleotide synthesis and microtubule dynamics, respectively, as well as pathways governing blast survival within the bone marrow niche. Critically, because our samples were obtained at diagnosis, before any exposure to chemotherapy, the differences we observe cannot be the result of drug-induced selection; they instead reflect a pre-existing cellular state, consistent with mechanisms of intrinsic chemoresistance [46].

Bioinformatics analysis revealed that biological pathways enriched with underexpressed proteins are associated with processes such as DNA replication, nuclear–cytoplasmic transport, and RNA degradation, as well as nucleic acid and cyclic organic compound metabolism, RNA processing, gene expression, and chromosomal organization, among others. All the cellular processes shown to be underexpressed are associated with pathways directly targeted by drugs such as MTX and VCR.

There are a limited number of reports on the use of omics data in B-ALL. In a multiomics study (DNA sequencing, RNA, RNA methylation array, and proteomics) of an adult patient with relapsed newly diagnosed B-cell precursor ALL, the enriched processes were related to chromatin modifiers, nucleotides, and steroid metabolism, highlighting candidates such as FPGS, mitochondrial cytosolic carboxypeptidase (AGBL), and zinc finger protein 483 (ZNF483), in addition to metabolic pathway proteins such as glucose-6-phosphate 1-dehydrogenase (G6PD), transketolase (TKT), glucose-6-phosphate isomerase (GPI), and glycerol-3-phosphate dehydrogenase (GPD) [47]. In this work, zinc finger protein 830 (ZNF830), a ZNF protein family member, was determined to be underexpressed (log2fc −2.5). ZNF830 promotes homologous recombination repair and survival in osteosarcoma cells in response to DNA, RNA, and nucleic acid metabolism damage [48]. The underexpression results show that tumor cells from nonresponder patients exhibit reduced nucleic acid synthesis and may resort to endocytic uptake of nucleotides from the microenvironment [49].

Proteins such as dehydrogenase/reductase SDR family member 6 (BDH2), CD8A, and PCNA were also shown to interact directly with MTX; however, BDH2 has been reported to function as a poor prognostic marker in acute myeloid leukemia (AML), with short overall survival, as it is associated with cellular anti-apoptosis [50]. When the interactions of underexpressed proteins with VCR were analyzed, QRICH1, MRPS16, BUB3, MGMT, SLC2A1, RPL6, TRPC4, and CD44 were identified. BUB3 participates in the assembly of the mitotic spindle; its overexpression has been associated with cancer progression and chemoresistance (mainly with microtubule-targeted drugs). However, in our study, its expression was decreased; this decrease has been associated with cellular senescence [51]. The overexpression of glucose transporter 1 (GLUT1 or SLC2A1) has been associated with chemoresistance in AML because it mediates glucose influx into tumor cells [52]; however, in this study, it was found to be underexpressed. RPL6 has been associated with multidrug chemoresistance in gastric and lung cancers, and inhibiting RPL6 decreases cell proliferation and migration [53]. The overexpression of TRPCs, such as 4 and 5, has been associated with drug resistance, as they are positively regulated by the multidrug resistance (MDR) transporter in breast cancer cells [54].

Nine overexpressed proteins involved in processes associated with tight junction structuring were identified, including the integrin subunit alpha 2b (ITGA2B) (log2, 2.7) and integrin subunit beta 3 (ITGB3) (log2, 2.9) (Figure 4). ITGA6 has been reported to regulate drug resistance in B-ALL, and its inhibition with an anti-human α6-blocking antibody (P5G10) induced apoptosis in vitro and sensitized cells to chemotherapy [55]. The overexpression of this protein is associated with the survival of immature B cells, as they bind to each other to survive. Additionally, in bone marrow, there is an environment rich in extracellular matrix components that activate signal transduction (integrin α4β1-fibronectin-VCAM-1), which confers chemoresistance in AML [56].

In the pancreatic cancer cell line PC-3, treatment with MTX reduced the expression of PCNA in the G1, S, and G2/M phases of the cell cycle, suggesting that PCNA is necessary for DNA synthesis [57]. With respect to TOP2, treatments such as anthracyclines, which intercalate into DNA and inhibit TOP2 [58], have been reported for leukemias, lymphomas, and solid tumors. Mutation or loss of PARP1 can promote resistance to PARP1 inhibitors by preventing PARP1 capture on DNA and suppressing the formation of DNA double-strand breaks (DSBs) [59].

Overexpressed proteins that interact with MTX include RDH11 and PRTN3. RDH11, which regulates reactive oxygen species (ROS) generation, has been associated with irinotecan resistance in melanoma cells [60]. In colorectal cancer, elevated serum expression of PRTN3 has been associated with poor progression-free survival (PFS) and increased angiogenesis, inhibiting the antiangiogenic capacity of bevacizumab [61].

The overexpressed proteins that interact with VCR include PTGS1, DOK2, and TLN2. It has been shown that inhibiting the expression of PTGS1, an oxidative stress modulator, enhances ROS production in non-small-cell lung cancer [62]. In chronic myeloid leukemia (CML), increased expression levels of PTGS1 and PTGS2 were associated with a favorable prognosis [63]. In ovarian cancer, decreased DOK2 expression was associated with treatment resistance, as apoptosis decreased [64]. Overexpression of TLN2 has been linked to cell migration and focal adhesion in breast cancer; however, it has not been linked to chemoresistance [65].

We next searched our dataset for canonical, literature-supported resistance mediators in ALL as internal positive controls (Supplementary Table S4). Several were recovered as DEPs in the expected direction: DHFR and ATIC (antifolate pathway) were underexpressed and MTHFR overexpressed; TUBB (vincristine target) was underexpressed; the efflux transporter ABCC1 was underexpressed; and the integrins ITGA2B and ITGB3 were overexpressed in nonresponders (Figure 4 and Figure 5d). Recovering these known markers confirms that our workflow detects genuine chemoresistance biology, reinforcing confidence in the novel candidates GSN and TYMP. Other established mediators (ABCB1, ABCG2, PAX5, IKZF1, CXCR4, HDAC1-3) were not detected. An expected limitation of single-shot DIA on unfractionated lysate is that it does not evidence that these mechanisms are absent.

For the first time, this work presents evidence of proteins associated with nonresponse in pediatric patients, identifies molecules associated with intrinsic chemoresistance, and enriches for processes highlighting actin cytoskeleton dynamics, metabolism, and nucleic acid synthesis during inherent chemoresistance. Both processes highlight proteins such as TYMP and GSN, key molecules involved in intrinsic chemoresistance, which can predict nonresponse in patients with pediatric B-ALL; both proteins were found to be overexpressed in MTX- and VCR-resistant patients. Based on the proteomic analysis of biopsies from patients who did not respond to first-line treatment, gelsolin (GSN) and thymidine phosphorylase (TYMP), two proteins involved in processes related to chemoresistance to these drugs, such as cytoskeleton dynamics [66] and nucleic acid metabolism [67], respectively, were selected.

GSN, as an actin-binding protein, can modify the cytoskeleton [68] and counteract the functional effect of VCR, which is to depolymerize the cytoskeleton [69], as GSN has been reported to interact with particular components of the three systems that make up the cytoskeleton, namely, nestin (intermediate filaments), Arp3 (actin cytoskeleton), and β-tubulin (microtubules), and possibly thereby modulate the dynamics of its structure by stabilizing it [70]. Recently, it has been shown that the effects of gelsolin on filament shearing, bending stiffness, and structural changes at acidic pH have implications for cell mechanics, suggesting that GSN plays a critical role in regulating the actin cytoskeleton and rapid remodeling [71].

In a phosphoproteomic study of primary acute lymphoblastic leukemia (ALL5) cells treated with VCR, Delgado et al., 2021 reported increased levels of microtubule-related proteins, including GSN [72]. However, the difference in GSN expression did not reach statistical significance. These authors concluded that VCR treatment increases the phosphorylation of cytoskeleton-associated proteins and thereby modulates their regulation [72]. In lymphoblasts, actin is usually disorganized or redistributed, and increased actin polymerization and depolymerization dynamics, which favor rapid proliferation, abnormal adhesion, migration, or tissue infiltration, are observed [73,74]. Microtubules have been suggested to play an essential role in the survival of primary ALL cells by affecting intracellular trafficking and mitosis in T lymphoblasts [75]. In response to this behavior, cytoskeleton regulatory proteins such as gelsolin, cofilin, and moesin are overexpressed, promoting cell survival [38].

Another protein selected was TYMP and its role in nucleotide metabolism, as a key enzyme of the pyrimidine salvage pathway [67], its contribution to angiogenesis [76], platelet activation [77], and inhibition of apoptosis [78], and the mechanistic rationale by which increased salvage capacity could offset the de novo nucleotide synthesis blockade imposed by MTX in nonresponding blasts. Also, TYMP has been reported to be overexpressed in different types of cancer, such as gastric carcinoma [79], non-small-cell lung cancer [80], colorectal cancer [81], cervical cancer [82], and bladder cancer [83].

Analysis of publicly available datasets from cBioPortal did not reveal statistically significant differences in GSN or TYMP mRNA expression between clinical groups. However, a trend toward increased GSN expression was observed in patients who experienced relapse. These findings should be interpreted with caution, as mRNA levels do not necessarily correlate with protein abundance or activity due to posttranscriptional and posttranslational regulation. In this context, the integration of bioinformatic analysis with proteomic data provides complementary insight by enabling comparisons between transcript-level patterns and experimentally determined protein expression, which is directly linked to biological function. In our study, a lack of response to first-line therapy was considered indicative of chemoresistance.

In contrast, relapse represents a clinically related outcome that the persistence of resistant cell populations may drive. Accordingly, the use of relapse-annotated datasets from cBioPortal was intended to provide a clinically relevant comparative framework. Nevertheless, these results should be interpreted cautiously and considered supportive rather than conclusive evidence.

A further limitation of this study concerns the genetic background of the cohort. It is well established that recurrent chromosomal translocations are the defining feature of the main molecular subtypes of pediatric B-ALL. The most notable examples of such translocations are ETV6::RUNX1, BCR::ABL1 and TCF3::PBX1. These translocations have been shown to influence the biology of treatment response. For example, relapses that are positive for ETV6::RUNX1 frequently acquire deletions in genes of the glucocorticoid signaling pathway, thereby acquiring glucocorticoid resistance [84]. In the present cohort, fusion status was not systematically recorded in the archived clinical files for all patients, and the sample size would not have supported a subtype-stratified analysis. Consequently, it was not possible to ascertain whether the proteomic variations observed between responders and nonresponders are independent of molecular subtype, or whether they are at least partly attributable to an unbalanced distribution of subtypes between the two groups. This should be regarded as a potential source of confounding rather than as evidence against our findings. The term ‘intrinsic (de novo) chemoresistance’ is employed to denote resistance already present in leukemic blasts at diagnosis, before any exposure to chemotherapy (the setting captured by our treatment-naive samples), as opposed to acquired resistance selected under therapeutic pressure. Intrinsic resistance is not confined to translocation-driven mechanisms; it may equally arise from baseline drug–efflux transporter expression, altered nucleotide and folate metabolism, cytoskeletal remodeling, or bone marrow microenvironmental cues. Future studies integrating cytogenetic and molecular subtype annotation with proteomic profiling in larger cohorts will be required to disentangle subtype-specific from subtype-independent determinants of intrinsic chemoresistance.

Another limitation of this study is that the overexpression of GSN and TYMP in nonresponders was established through LC-MS/MS-based proteomic quantification alone, without orthogonal validation at the protein level. Individual per-patient abundance values (Supplementary Table S2) support the internal consistency and transparency of this quantitative approach; however, mass spectrometry-derived abundance measurements have not been corroborated by an independent immunodetection method in this study. Due in part to the limited quantity of protein obtainable per patient: bone marrow aspirates from pediatric ALL patients are a finite, non-renewable clinical resource, and the amount of material available after LC-MS/MS processing constrained the feasibility of performing additional orthogonal assays within the scope of this study. As a future perspective, orthogonal validation by Western blot or immunocytochemistry (ICC), performed under a standardized and adequately replicated protocol in an independent, adequately powered patient cohort with sufficient sample material, would strengthen confidence in GSN and TYMP as candidate predictors of chemoresistance in pediatric B-ALL and represents a desirable next step before their consideration for clinical application.

## 4. Materials and Methods

### 4.1. Patient Samples and Ethical Statements

A case–control study was performed on bone marrow aspirates cryopreserved in the Biobank of the Laboratory of Molecular Biomedicine of the Autonomous University of Guerrero [85]. This study was conducted in accordance with the guidelines of the Declaration of Helsinki and approved by the Institutional Ethics Committee of the Instituto Estatal de Cancerología “Dr. Arturo Beltrán Ortega” (protocol code DGIECAN/CCHGC/CEI/PRO-039-2024; approval date: 25 April 2024). Written informed consent was obtained from the legal guardians of all participants prior to inclusion. All clinical data were anonymized before analysis.

Cases and controls were cryopreserved between 2012 and 2019. They consisted of 20 bone marrow aspirate samples from patients diagnosed with B-ALL according to the French–American–British morphological classification, cytochemical staining properties, and blast immunophenotyping. The diagnosis, subclassification into B or T, and treatment regimen, as well as data on complete remission, relapse, and poor outcomes, were previously described [17]. Patients were aged between 2 and 14 years (responders) and 1 and 17 years (nonresponders) at diagnosis (median in both groups, 8 years); the upper eligibility limit was 17 years and 11 months. All were newly diagnosed with B-ALL, untreated at the time of sampling, and had documented clinical information on response or nonresponse to treatment (the patient’s relapse determined this classification). Clinical data (age, sex, treatment response, among others) were obtained from the clinical records (Supplementary Table S1). The cases included 10 patients who were diagnosed with B-ALL and who were nonresponders to induction treatment with vincristine (1.4 mg/m^2^) and methotrexate (8 mg/m^2^).

Patient samples were included based on the following criteria: (a) proteins cryopreserved in the organic phase derived from TRIzol processing of bone marrow aspirates from pediatric patients (1–17 years old) diagnosed with B-cell acute lymphoblastic leukemia and treated with first-line methotrexate and vincristine; and (b) availability of complete clinical information, including patient identification codes and treatment response classification based on minimal residual disease (MRD) percentage. Samples were excluded if they did not meet the minimum protein concentration required for downstream processing (<50 µg of total protein after TRIzol-based extraction) or if they failed quality control during LC–MS/MS analysis (inadequate chromatographic separation). Of the 20 samples initially selected (10 per group), one did not reach the ≥50 µg threshold, and two showed inadequate chromatographic separation; these three samples were excluded, leaving 17 samples in the final analysis. No samples were excluded because of missing patient identification, as all samples were anonymized and assigned unique codes before analysis in accordance with ethical guidelines.

### 4.2. Protein Extraction from Patient Samples

Cells were lysed with TRIzol–chloroform (15596018, Thermo Fisher, Carlsbad, CA, USA) according to the manufacturer’s instructions, as described in the protein extraction protocol. Washes were performed with 0.3 M guanidine hydrochloride (Sigma–Aldrich 50-01-1, St. Louis, MO, USA) in 95% ethanol. The proteins were subsequently resuspended in 1% SDS.

### 4.3. Digestion, Reduction, and Alkylation

50 µg of the precipitated protein extract was resuspended in 50 µL of Tris (pH 8.8). Once dissolved, reduction was carried out with dithiothreitol (DTT) to a final concentration of 100 mM, and the mixture was incubated at 56 °C for 30 min, followed by alkylation with iodoacetamide to a final concentration of 200 mM and incubation at RT for 30 min in the dark. The modified proteins were solubilized in 50 mM ammonium bicarbonate and 0.5% deoxycholate, and 1 µg of modified porcine trypsin (purity grade) was added. Digestion was carried out at 37 °C for 16 h. Detergent extraction was performed with ethyl acetate and trifluoroacetic acid at a final concentration of 0.5%. Peptides were dried in a SpeedVac and stored at −80 °C until analysis.

### 4.4. Protein Quantification

Total extracts were quantified using the Pierce 660 nm method (Thermo Fisher Scientific, Waltham, MA, USA) in a microplate according to the manufacturer’s protocol. Briefly, 10 µL of each bovine serum albumin standard (125, 250, 500, 750, 1000, 1500 and 2000 µg/mL) and 10 µL of the test samples and the blank (SDS-Tris 1% lysis buffer) were added in triplicate, after which 150 µL of the protein assay reagent was added to each well. The readings were performed on a Multiskan FC plate reader (Thermo Fisher Scientific, MA, USA) with a previous shaking at medium speed for 5 min. The absorbance was measured at λ = 660 nm. The protein concentration was determined prior to reduction, alkylation, and enzymatic digestion. In addition, the peptide concentration was measured using a NanoDrop spectrophotometer 2000c Spectrophotometer (Thermoscientific, Whaltham, MA, USA) before LC–MS/MS analysis.

### 4.5. Analysis by LC–MS/MS

#### 4.5.1. Identification/Quantification of Peptides/Proteins

As previously described [86], 1 μg of the peptide mixtures was analyzed using a liquid chromatography–mass spectrometry system composed of a Dionex Ultimate 3000 RSLCnano UPLC (Thermo Fisher Scientific, Waltham, MA, USA) coupled to a Q-Exactive HF-X mass spectrometer (Thermoscientific, Whaltham, MA, USA). The mass spectrometer was operated in positive mode, and the data were acquired using an in-house-developed variable-window data-independent acquisition method. Peptides were loaded and desalted on an Acclaim PepMap100 C18 trap column (Thermoscientific, Whaltham, MA, USA) (3 µm, 100 Å, 75 µm i.d. × 2 cm, nanoViper) with aqueous 0.1% TFA at a flow rate of 5 µL/min for 6 min. Peptide separation was performed in-line, connecting the trap column to an EASY-Spray RSLC C18 analytical column (Thermoscientific, Whaltham, MA, USA) (2 µm, 100 Å, 75 µm i.d. × 50 cm) at a flow rate of 300 nL/min. The trap and analytical columns were set to 35 °C and 60 °C, respectively. Peptides were separated using a nonlinear gradient over 60 min, starting from 2% to 27% buffer B in 55 min, then from 27% to 35% and from 35% to 55% over 2 min each, and finally reaching 90% buffer B in 1 min. The acquisition method consisted of a full acquisition cycle comprising 54 variable-width MS2 scans and 3 MS1 scans over the mass range 385–1460 m/z. The MS2 window widths were determined from the empirical distribution of signals from data-dependent acquisition (DDA) runs with similar samples, with a 1 Da overlap between adjacent windows. The parameters for MS1 were as follows: resolution of 120,000 (@ 200 m/z), AGC target of 3 × 10^6^, and maximum injection time of 50 ms. For MS2, the resolution was set to 30,000, with an AGC target of 1 × 10^6^, automatic maximum injection time, and an NCE of 28.

Each peptide digest (1 µg on column) was analyzed in a single LC–MS/MS injection; no technical replicates were acquired. Instrument performance was monitored throughout the batch using dedicated quality-control samples, and the high run-to-run reproducibility of the DIA workflow, verifiable from the deposited raw files (PXD074132), makes repeated injections of the same digest quantitatively redundant. Consequently, all differential expression estimates rest on biological replication across the responder and nonresponder groups.

#### 4.5.2. Bioinformatic Analysis for Peptide and Protein Identification

Raw DIA files were processed with DIA-NN v1.8.1 [87] for precursor search and fragment identification using two-dimensional peak positioning (retention time and precursor m/z), with spectral library prediction via neural networks. The key search parameters included protein inference = “Genes”, quantification = “Robust LC (High precision)”, cross-run normalization = “RT-dependent”, with MBR, isotopologue use, and heuristic protein inference enabled. Searches were performed against the UniProt human proteome (UP000005640, released 27 July 2024) using trypsin digestion, fixed carbamidomethylation of cysteines, and variable methionine oxidation. Data harmonization was performed with MS-DAP v1.1 [88], and only samples with ≥3 detected peptides were retained. Normalization combined variance stabilizing normalization (VSN) [89] and the “modebetween_protein” method. Differential expression between responders and nonresponders was assessed with MS-EmpiRe v0.1.0 [90], applying contrast-specific peptide filtering to maximize the number of peptides available per comparison. A total of 31,399 peptides across 4365 proteins were included in this contrast. Differentially expressed proteins (DEPs) were defined by a q value ≤ 0.05 and a |log_2_ fold change| ≥ 0.85 (bootstrap-estimated). PCA was computed on peptides consistently detected within each group to assess global sample variation while minimizing the influence of sporadically detected or low-abundance features.

### 4.6. Bioinformatic Analysis

Differentially expressed proteins (DEPs) identified by LC–MS/MS were analyzed by their Universal Protein Resource (UNIPROT) identification code (http://www.uniprot.org) in databases such as Reactome (http://www.reactome.org/), Gene Ontology (http://geneontology.org/), Panther (http://pantherdb.org/), The Human Protein Atlas (http://www.proteinatlas.org/), Gene Expression Profiling Interactive Analysis (GEPIA) (http://gepia.cancer-pku.cn/) and the Search Tool for the Retrieval of Interacting Genes/Proteins (STRING) (https://string-db.org/) to detail their functions, possible interactions with other proteins and their role in chemoresistance, considering the mechanism of cytoskeleton-binding proteins [accessed 12 August 2025]. To perform biological pathway enrichment analysis, ShinyGo 0.85 software (https://bioinformatics.sdstate.edu/go/) was used [last accessed 22 August 2025]. The Cytoscape 2.10.4 platform was used for editing protein–protein and protein–drug interactions.

### 4.7. TCGA Dataset

The Cancer Genome Atlas Research Network of Lymphoid Neoplasm (TCGA-B lymphoblastic leukemia/lymphoma) was used to visualize the clinical data. The cBioPortal resource (https://www.cbioportal.org/) was reviewed, selecting lymphoid cancers, comprising 24 databases, from which the dataset ‘’Pediatric Acute Lymphoid Leukemia—Phase II, (TARGET, 2018) [Accessed 22 September 2025] was selected. This dataset contains information on 1551 patients and 1978 samples. Among these patients, 1261 had an unknown type of therapy, and 206 patients were treated with chemotherapy alone. The graphs of the percentage of relapse in bone marrow, which is related to chemoresistance, were reviewed. Once the selection filters were established, the differences in the expression (mRNA) of GSN and TYMP in patients with and without relapse in bone marrow were identified.

### 4.8. Statistical Analysis of GSN and TYMP Protein Abundance

Per-patient relative abundance values for GSN and TYMP, derived from the LC-MS/MS quantification described in Section 4.5, were extracted for each sample included in the responder and nonresponder groups and are reported in full in Supplementary Table S2. Normality of the abundance distributions was assessed for each protein and group using the Shapiro–Wilk test prior to group comparison. GSN abundance values did not follow a normal distribution in either the responder or nonresponder group; accordingly, differences in GSN abundance between groups were assessed using the Mann–Whitney U test. TYMP abundance values were normally distributed in both groups, with no significant difference in variance between groups (F test, *p* = 0.2985); accordingly, differences in TYMP abundance between groups were assessed using an unpaired Student’s *t*-test. A *p* < 0.05 was considered statistically significant for both comparisons. Statistical analysis and graphical representation (Figure 5c) were performed using GraphPad Prism v8.0.1. Full normality and variance test results supporting these statistical choices are provided in Supplementary Table S3.

## 5. Conclusions

This work presents evidence of proteins GSN and TYMP that may be related to nonresponse to chemotherapeutics administered during the induction phase of pediatric B-ALL treatment, reinforcing actin/tubulin-based cytoskeleton remodeling and nucleic acid synthesis as processes that could be considered hallmarks of chemoresistance in acute lymphoblastic leukemia. This pattern is supported by quantitative LC-MS/MS analysis, performed under a single standardized analytical workflow, with individual per-patient values provided in Supplementary Table S2. GSN and TYMP therefore represent candidate targets warranting validation by orthogonal methods in an independent, adequately powered patient cohort.

## Data Availability

The mass spectrometry proteomics data have been deposited to the ProteomeXchange Consortium (https://proteomecentral.proteomexchange.org) via the iProX partner repository [91,92] with the dataset identifier PXD074132. Per-sample GSN and TYMP abundance values underlying Figure 5c are additionally provided in Supplementary Table S2.

## Supplementary Materials

The following supporting information can be downloaded at: https://www.mdpi.com/article/10.3390/ph19081167/s1. Table S1: Clinical data of patients with ALL; Table S2: Raw protein abundance data obtained by HPLC-MS/MS analysis. Table S3: Statistical analysis of per-patient GSN and TYMP abundance determined by LC-MS/MS. Table S4: Curated panel of canonical mediators of methotrexate and vincristine resistance and their detection status in the proteomic dataset.

## Abbreviations

The following abbreviations are used in this manuscript:

B-ALL	Acute Lymphoblastic Leukemia
BCR	B-cell receptor
TYMP	Thymidine phosphorylase
GSN	Gelsolin
DEP’s	Differential Expressed Proteins
AML	Acute Myeloid Leukemia
ROS	Reactive Oxygen Species
UNIPROT	Universal Protein Resource
GEPIA	Gene Expression Profiling Interactive Analysis
STRING	Search Tool for the Retrieval of Interacting Genes/Proteins
TCGA	The Cancer Genome Atlas
ETV6	ETS variant Transcription Factor 6
BCR	Breakpoint Cluster Region Protein
ABL1	Tyrosine-Protein Kinase ABL1
PAX5	Paired Box Protein Pax-5
ABCG2	ATP Binding Cassette Subfamily G Member 2
MDR1	ATP-Dependent Translocase ABCB1
CASP3	Caspase 3
CASP8	Caspase 8
BCL-XL	B-Cell Lymphoma-Extra Large
HDAC3	Histone Deacetylase 3
ITGA4	Integrin Alpha-4
ITGA6	Integrin Alpha-6
CD44	CD44 antigen
QRICH1	Transcriptional Regulator QRICH1
MRPS16	Small ribosomal subunit protein bS16m
BUB3	Mitotic Checkpoint Protein BUB3
SLC2A1	Solute Carrier Family 2
RPL6	Large Ribosomal Subunit Protein El6
TRPC4	Short Transient Receptor Potential Channel 4
MGMT	Methylated-DNA-Protein-Cysteine Methyltransferase
BDH2	Dehydrogenase/Reductase SDR Family Member 6
CD8A	T-Cell Surface Glycoprotein CD8 Alpha Chain
PCNA	Proliferating Cell Nuclear Antigen
TOP2B	DNA Topoisomerase 2-Beta
PARP1	Poly [ADP-Ribose] Polymerase 1
RDH11	Retinol Dehydrogenase 11
PRTN3	Proteinase 3
PTGS1	Prostaglandin G/H Synthase 1
DOK2	Docking Protein 2
TLN2	Talin-2
SLC19A1	Reduced Folate Transporter
CYP3A4	Cytochrome P450 Family 3 Subfamily A Member 4
CYP3A5	Cytochrome P450 Family 3 Subfamily A Member 5
DHFR	Dihydrofolate Reductase
FPGS	Folylpolyglutamate Synthase
GGH	Gamma-Glutamyl Hydrolase
MTHFR	Methylenetetrahydrofolate Reductase (NADPH)
ATIC	Bifunctional Purine Biosynthesis Protein ATIC
TYMS	Thymidylate Synthase
GART	Trifunctional Purine Biosynthetic Protein Adenosine-3
ABCC1	ABC Transporter C Family Member 1
TUBB	Tubulin Beta Chain
AGBL	Mitochondrial Cytosolic Carboxypeptidase
ZNF483	Zinc Finger Protein 483
G6PD	Glucose-6-Phosphate 1-Dehydrogenase
TKT	Transketolase
GPI	Glucose-6-Phosphate Isomerase
GPD	Glycerol-3-Phosphate Dehydrogenase
ZNF830	Zinc Finger Protein 830
MDR	Multidrug Resistance
ITGA2B	Integrin Subunit Alpha 2b
ITGB3	Integrin Subunit Beta 3
P5G10	Anti-Human A6-Blocking Antibody
DTT	Dithiothreitol
DIA-NN	Data-Independent Acquisition Neural Networks
DDA	Data-Dependent Acquisition
MBR	Run-To-Run Matching
FDR	False Discovery Rate
MaxLFQ	Maximally Label-Free Quantification
SDS-PAGE	Sodium Dodecyl Sulfate Polyacrylamide Gel Electrophoresis
TBS-T	Tris-Buffered Saline With Tween-20
GAPDH	Glyceraldehyde-3-Phosphate Dehydrogenase
HRP	Horseradish Peroxidase
