# Supplementary material for: Proteomic Profiling of Bone Marrow Aspirates from Patients with Methotrexate- and Vincristine-Resistant B-Cell Acute Lymphoblastic Leukemia: A Retrospective Analysis
Source: Pharmaceuticals (Basel). 2026 Jul 26;19(8):1167. doi: 10.3390/ph19081167 (PMC13516805; doi:10.3390/ph19081167)
Supplement: Supplementary file 1 [file pharmaceuticals-19-01167-s001.zip › Supplementary table ST3.pdf]

GSN	
Responders	Nonresponders
22.45246636	24.03898096
21.64393555	23.53281335
23.3498484	24.37004875
19.08292079	23.68703134
20.31327849	23.78315149
19.83809321	21.60623079
22.94981941	24.05926206
20.64802487	23.47900764
	23.470221

TYMP	
Responders	Nonresponders
19.6941235	22.35935181
16.93865071	22.20975896
20.11871948	23.01922506
18.28810603	22.36229952
18.56420715	22.68216943
19.60352518	24.16587616
20.70389976	23.08172123
17.9112972	21.31132594
	21.55002686

GSN

	Normality and Lognormality Tests<br>Tabular results	A<br>Responders	B<br>Nonresponders
1	**Test for normal distribution**		
2	**Anderson-Darling test**		
3	A2*	0.2176	0.9369
4	P value	0.7589	0.0098
5	Passed normality test (alpha=0.05)?	Yes	No
6	P value summary	ns	**
7			
8	**D'Agostino & Pearson test**		
9	K2	1.366	15.20
10	P value	0.5051	0.0005
11	Passed normality test (alpha=0.05)?	Yes	No
12	P value summary	ns	***
13			
14	**Shapiro-Wilk test**		
15	W	0.9498	0.7576
16	P value	0.7089	0.0066
17	Passed normality test (alpha=0.05)?	Yes	No
18	P value summary	ns	**
19			
20	**Kolmogorov-Smirnov test**		
21	KS distance	0.1594	0.3446
22	P value	>0.1000	0.0028
23	Passed normality test (alpha=0.05)?	Yes	No
24	P value summary	ns	**
25			
26	**Number of values**	8	9
27			

TYMP

	Normality and Lognormality Tests<br>Tabular results	A<br>Responders	B<br>Nonresponders
1	**Test for normal distribution**		
2	**Anderson-Darling test**		
3	A2*	0.1969	0.2364
4	P value	0.8291	0.7023
5	Passed normality test (alpha=0.05)?	Yes	Yes
6	P value summary	ns	ns
7			
8	**D'Agostino & Pearson test**		
9	K2	0.3851	0.9323
10	P value	0.8248	0.6274
11	Passed normality test (alpha=0.05)?	Yes	Yes
12	P value summary	ns	ns
13			
14	**Shapiro-Wilk test**		
15	W	0.9676	0.9590
16	P value	0.8783	0.7876
17	Passed normality test (alpha=0.05)?	Yes	Yes
18	P value summary	ns	ns
19			
20	**Kolmogorov-Smirnov test**		
21	KS distance	0.1901	0.1471
22	P value	>0.1000	>0.1000
23	Passed normality test (alpha=0.05)?	Yes	Yes
24	P value summary	ns	ns
25			
26	**Number of values**	8	9
27			

**Protein**	**Group**	**Shapiro-Wilk W**	**Shapiro-Wilk p**	**Test F**	**Statistical test**	**Significantly different (p < 0.05)**
**GSN**	Responders	0.9498	0.7089		Mann-Whitney U	***p*=0.0010**
**GSN**	Nonresponders	0.7576	0.0066		Mann-Whitney U	
**TYMP**	Responders	0.9676	0.8783	0.2985	Unpaired t-test	***p*<0.0001**
**TYMP**	Nonresponders	0.9590	0.7876		Unpaired t-test	
